# Voltage-Sensitive Dye versus Intrinsic Signal Optical Imaging: Comparison of Tactile Responses in Primary and Secondary Somatosensory Cortices of Rats

**DOI:** 10.3390/brainsci11101294

**Published:** 2021-09-29

**Authors:** Ichiro Takashima, Riichi Kajiwara

**Affiliations:** 1 Human Informatics and Interaction Research Institute, National Institute of Advanced Industrial Science and Technology (AIST), Tsukuba 305-8568, Japan; 2 Department of Electronics and Bioinformatics, School of Science and Technology, Meiji University, Kawasaki 214-8571, Japan; rdkaji@meiji.ac.jp

**Keywords:** optical imaging, somatosensory cortex, whisker, hemodynamics, neurovascular coupling

## Abstract

Studies using functional magnetic resonance imaging assume that hemodynamic responses have roughly linear relationships with underlying neural activity. However, to accurately investigate the neurovascular transfer function and compare its variability across brain regions, it is necessary to obtain full-field imaging of both electrophysiological and hemodynamic responses under various stimulus conditions with superior spatiotemporal resolution. Optical imaging combined with voltage-sensitive dye (VSD) and intrinsic signals (IS) is a powerful tool to address this issue. We performed VSD and IS imaging in the primary (S1) and secondary (S2) somatosensory cortices of rats to obtain optical maps of whisker-evoked responses. There were characteristic differences in sensory responses between the S1 and S2 cortices: VSD imaging revealed more localized excitatory and stronger inhibitory neural activity in S1 than in S2. IS imaging revealed stronger metabolic responses in S1 than in S2. We calculated the degree of response to compare the sensory responses between cortical regions and found that the ratio of the degree of response of S2 to S1 was similar, irrespective of whether the ratio was determined by VSD or IS imaging. These results suggest that neurovascular coupling does not vary between the S1 and S2 cortices.

## 1. Introduction

Two optical imaging methods are commonly used to monitor brain activity: voltage-sensitive dye (VSD) and intrinsic signals (IS) [1,2]. Although both methods produce similar brain activity maps, the information they represent is fundamentally different. VSD imaging is a direct representation of the excitatory and inhibitory neuronal activities, whereas IS imaging represents hemodynamic and metabolic changes in brain tissue, similar to the signals depicted by functional magnetic resonance imaging (fMRI). In a previous study, we compared VSD and IS imaging optical maps of the rat barrel cortex in response to whisker stimulation and demonstrated that IS maps before the changes in cerebral blood volume were nearly identical in location and size to VSD maps of the excitatory neural responses [3]. A few later studies described the characteristics and relationships between simultaneously performed VSD and IS imaging maps [4,5,6]. However, all previous studies, including ours, were conducted on the somatosensory cortex; therefore, it is unclear whether the reported findings also apply to other brain regions. In addition, even within the somatosensory cortex, whether the primary (S1) and secondary (S2) somatosensory areas demonstrate a similar relationship between VSD and IS maps has not been investigated.

The interpretation of fMRI data remains ambiguous because fMRI signals are an indirect measure of neural activity and knowledge of the underlying neurovascular coupling mechanism is incomplete [7]. As an example, it is assumed that neurovascular coupling is spatially invariant; however, relatively recent studies have identified differences in neurovascular coupling across brain regions [8,9,10]. These studies compared fMRI and electrophysiologic data and identified regional variability in neurovascular coupling, e.g., between cortical and subcortical regions, and between primary and secondary cortical areas. The combination of fMRI and electrophysiology is a promising approach to elucidate neurovascular coupling properties. For the superficial cortex, IS and VSD imaging provide similar information and better spatiotemporal resolution compared to fMRI and electrophysiology, respectively. Although the combination of IS and VSD imaging can be used to characterize cortical neurovascular coupling, it has only been used in a small number of studies [11].

In the present study, we performed VSD and IS imaging of the S1 and S2 cortices in rats to create optical maps of whisker-evoked responses. The first objective was to describe the characteristics of VSD and IS imaging representation of brain activity in the two sensory areas. The second was to compare the ratio of S1 and S2 sensory responses between VSD and IS imaging. We hypothesized that if neurovascular coupling is invariant between the S1 and S2 cortices, the ratio of S1 and S2 sensory responses obtained from the two imaging methods, i.e., the ratio of the magnitude of neural responses (S2/S1 ratio from VSD maps) and the ratio of the magnitude of metabolic responses (S2/S1 ratio from IS maps), would be the same. Our results clearly show spatiotemporal differences in sensory responses from the S1 and S2 cortices, with the most characteristic difference being the strong inhibitory neural activity seen from the S1 cortex. Furthermore, a simple model suggested that if the hemodynamic component resulting from S1 inhibitory neural activity is taken into account, neurovascular coupling does not vary between S1 and S2.

## 2. Materials and Methods

### 2.1. Animals

All experiments were approved by the Committee on Animal Care and Use and the Ethical Committee of the National Institute of Advanced Industrial Science and Technology. Experiments were performed on seven male Wistar rats (200–230 g; SLC Inc., Shizuoka, Japan). The rats were housed in cages under a 12 h light/dark cycle with food and water provided ad libitum.

### 2.2. Surgery for Optical Imaging

Each animal was anesthetized with an intraperitoneal injection of ketamine (80 mg/kg) and xylazine (10 mg/kg). The animals were positioned in a stereotaxic apparatus (SR-50R-HT; Narishige, Tokyo, Japan), and a craniotomy (5 × 5 mm) was performed over the left somatosensory cortex. The dura mater was removed, and a well of dental acrylic was built around the exposed cortex. The cortical surface was stained with a fluorescent dye (RH-795; Thermo Fisher Scientific, Waltham, MA, USA) dissolved in artificial cerebrospinal fluid (ACSF; 125 mM NaCl, 2.5 mM KCl, 2 mM CaCl_2_, 1.25 mM MgSO_4_, 1.25 mM NaH_2_PO_4_, 22 mM NaHCO_3_, and 10 mM glucose; pH 7.3–7.4) at 0.6 mg/mL [12]. After staining for 40 min, the unbound dye was thoroughly washed out with ACSF. Thereafter, the well was filled with ACSF and sealed with a glass coverslip. Supplemental injections of ketamine and xylazine were used to maintain a constant level of anesthesia, as indicated by respiratory rate, heart rate, corneal reflex, and foot withdrawal reflex. The body temperature was kept at 37 °C using a thermostatically controlled heating pad.

### 2.3. Whisker Stimulation

Whiskers were trimmed to 20 mm, and the end of an electromechanical actuator probe (P1001; TDK Corp., Tokyo, Japan) was placed orthogonal to the shaft of whisker B1, 15 mm from the skin surface [3]. The whisker was deflected once using a single pulse delivered to the actuator, displacing it 0.5 mm for 10 ms. The initial movement was in a caudal to rostral direction, and whisker deflection did not move the adjacent whiskers.

### 2.4. Optical Imaging

**Figure 1:**
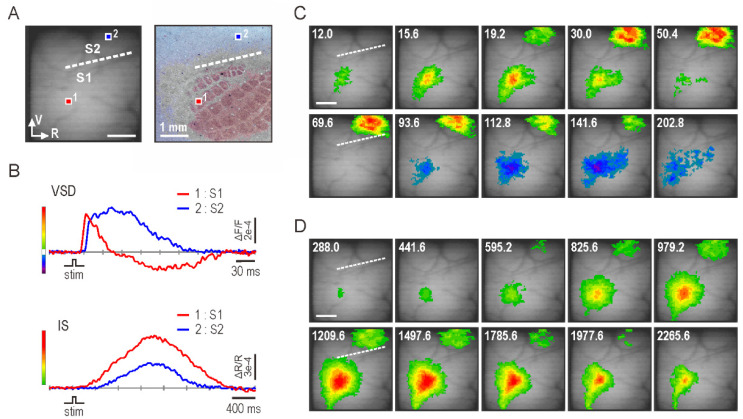
Whisker-evoked somatosensory responses visualized by voltage-sensitive dye (VSD) and intrinsic signal (IS) optical imaging. (**A**) Surface image of the recorded cortical area (left) and the underlying barrel architecture confirmed by CO staining (right). Dotted lines show the border between the primary (S1) and secondary (S2) somatosensory cortices. (**B**) Representative optical signals recorded by VSD (top panel) and IS (bottom panel) imaging. Red traces show VSD (top) or IS (bottom) optical signals detected by pixels in S1 (small red rectangle 1 in A). Meanwhile, blue traces represent optical signals detected in S2 (small blue rectangle 2 in A). The dye signal reports the membrane depolarization (or hyperpolarization) of ensemble of cortical neurons as an upward (or downward) signal change. Upward change in the trace of IS represents an increase in cerebral blood volume and blood deoxyhemoglobin concentration. (**C**) Spatiotemporal dynamics of neural activity revealed by VSD imaging. VSD signals (B, top panel) were color coded and superimposed on background cortical image. Warm and cold colors represent excitatory and inhibitory neural activities, respectively. Whisker was stimulated at 0 ms; time after stimulation is indicated in each image (ms). (**D**) Spatiotemporal spread of metabolic activity as visualized by IS imaging. Metabolic activity was encoded in pseudocolor, using the intrinsic optical signals (B, bottom panel). S1: primary somatosensory cortex, S2: secondary somatosensory cortex, R: rostral, and V: ventral. Scale bars, 1 mm.

Optical signals from the exposed cortex were recorded using a tandem-type epifluorescence microscope and a metal oxide semiconductor-based monolithic array camera (128 × 128 pixels), both of which were developed in our laboratory [13,14]. A cortical area of about 4.2 × 4.2 mm was imaged with the camera attached to the microscope (Figure 1). The rat’s head was rotated by approximately 40–50° to ensure that the camera axis was perpendicular to the brain surface. Light from a tungsten-halogen lamp was guided to a removable filter cube by an optical fiber. The filter cube used for VSD imaging consisted of an excitation light filter (535 nm), dichroic mirror (580 nm), and long-pass filter (600 nm). The filter cube used for IS imaging consisted of an interference filter (610 nm) for brain illumination and a half mirror. Optical images were acquired at a rate of 1.2 ms/frame (VSD) or 19.2 ms/frame (IS). Data from 16 trials with an interstimulus interval of 24 s were averaged.

The experiment was performed with VSD imaging followed by IS imaging. Throughout the optical imaging experiments, particularly before VSD imaging and after IS imaging, we recorded epicortical potentials to whisker stimulation using a silver ball electrode and confirmed that no changes occurred in the size or shape of the surface potentials [3].

### 2.5. Analysis of Imaging Data

**Figure 2:**
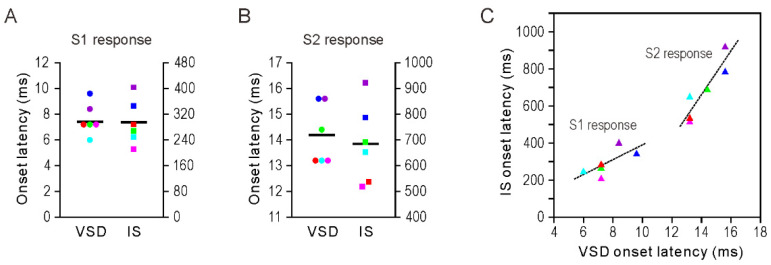
Onset latency of S1 and S2 responses. (**A**) Onset latency of S1 responses obtained with voltage-sensitive dye (VSD) and intrinsic signal (IS) imaging. Each circle and square represents one rat, and each color represents the same individual (n = 6). Horizontal bars represent mean. (**B**) Similar information as in A, but for S2 responses. (**C**) Correlation of onset latency of somatosensory responses recorded by VSD and IS imaging. Each triangle represents one animal, and its color corresponds to that of A and B. Broken lines represent regression lines for S1 and S2 responses.

**Figure 3:**
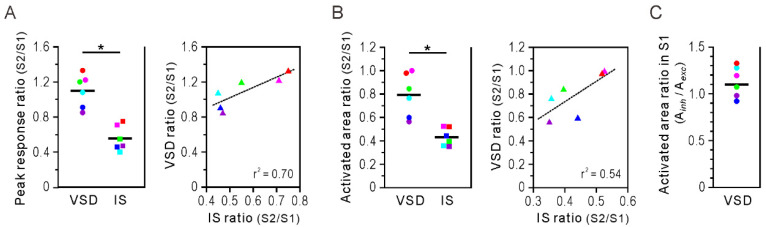
Amplitude and extent of S1 and S2 responses. (**A**) Peak response ratio. Ratio of the maximum amplitude value of the S2 response to that of the S1 response was determined for voltage-sensitive dye (VSD) and intrinsic signal (IS) imaging results (left panel). Each circle and square represents one animal (n = 6). Peak response ratio S2/S1 was significantly larger in VSD imaging than IS imaging (* *p* < 0.05, Wilcoxon signed-rank test, n = 6). The relationship between S2/S1 ratios obtained by VSD and IS imaging is shown in the right panel. The color of each triangle corresponds to an individual of the same color on the left panel. Broken line represents the regression line of best fit. r^2^: coefficient of determination. (**B**) Activated area ratio. Ratio of the maximum activation area of the S2 response to that of the S1 response was calculated for VSD and IS imaging (left panel). The activated area ratio S2/S1 was significantly larger in VSD imaging than IS imaging (* *p* < 0.05, Wilcoxon signed-rank test, n = 6). The right panel shows the relationship between the S2/S1 ratios obtained by VSD and IS imaging. Each triangle represents one animal. In the analysis of VSD imaging data, peak amplitude and area of maximum activation were determined only for excitatory neural activity. (**C**) Area ratio of inhibitory/excitatory regions in S1. Based on VSD imaging results, maximum activation areas of inhibitory (A*_inh_*) and excitatory (A*_exc_*) neural activity were assessed, and ratios were plotted for individual rats. Horizontal bars represent mean.

**Figure 4:**
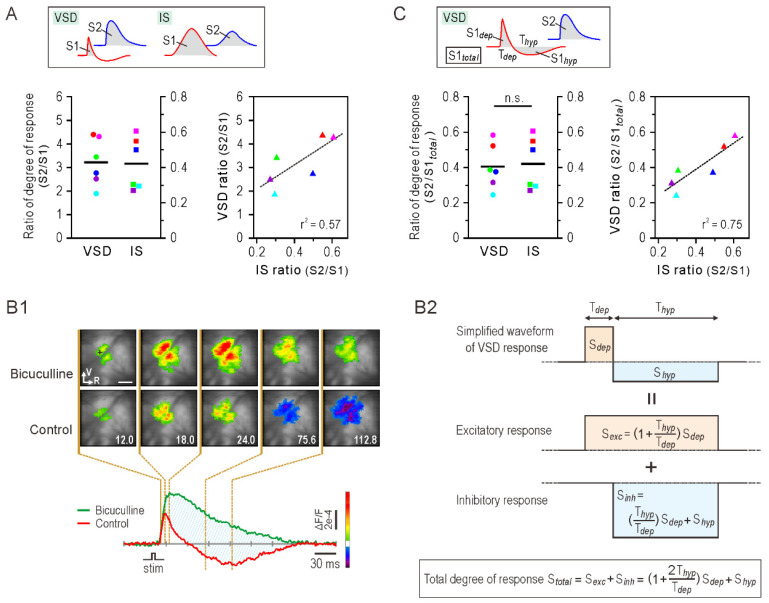
Analysis of the degree of sensory responses in S1 and S2 cortices. (**A**) Ratio of degree of response between S1 and S2. The degree of response was calculated as the time-integrated value of the optical signals (gray area in the top panel) selected from each response center. Note that the signal component representing cortical inhibition (downward deflection of the S1 response in VSD imaging) was excluded. The ratio of the degree of response between S1 and S2 responses was determined for VSD and IS imaging (bottom left panel). Horizontal bars are the mean values (n = 6). Note that the S2/S1 ratio reported by VSD imaging was about one digit larger than that by IS imaging. Bottom right panel shows the relationship between the degree of response ratios (S2/S1) obtained by VSD and IS imaging. Circles, squares, and triangles of the same color indicate the same individual. Broken line represents the regression line of best fit. r^2^: coefficient of determination. (**B1**) VSD imaging of S1 responses to whisker stimulation before and after blocking inhibitory activity. Optical recordings were taken before and after application of bicuculline (100 µM), which was dropped over the exposed cortical surface and washed away after 3 min. Time-series images show spatiotemporal spread of neural activity at representative time points before (lower row) and after (upper row) bicuculline. Red (control) and green (bicuculline) traces represent the time course of the VSD signals recorded by pixels indicated by a cross in the upper left image. Hatched area indicates the degree of response masked by GABA_A_ receptor-mediated inhibition. Poststimulus time (ms) is indicated in each image. R: rostral, V: ventral. Scale bars, 1 mm. (**B2**) Analytical model of cortical degree of response, taking into account inhibitory neural activity. Typical VSD signal trace in the S1 shows an excitatory-inhibitory sequence: a short, transient upward signal component followed by a long-lasting, downward signal component (B1, control). Therefore, we simplified the VSD signal trace as shown in the top row waveform, where T*_dep_* and T*_hyp_* are the duration of depolarizing and hyperpolarizing signal components, respectively, and S*_dep_* and S*_hyp_* are the time integrals of each. The simplified VSD response was decomposed into excitatory (middle) and inhibitory (lower) responses. S*_exc_* and S*_inh_* represent the time integrals of the excitatory and inhibitory responses, respectively, and the sum of the two is the total degree of cortical response (S*_total_*, equation in the bottom box). (**C**) Same analysis as in A, but inhibitory activity was considered for VSD data (IS results are identical to A). The value S1*_total_*, calculated using S1*_dep_* and S1*_hyp_* (as described in B2), was used for the degree of response of S1 for VSD imaging (top panel). The ratio of the degree of response S2/S1*_total_* was not significantly different between VSD and IS imaging (*p* = 0.674, Wilcoxon signed-rank test, n = 6, bottom left panel). Compared to the result in A, regression analysis showed a better coefficient of determination between ratios obtained from both imaging results (bottom right panel).

After binning 2 × 2 pixels into one super-pixel each, we identified the super-pixel with the greatest amplitude change in the optical signal. This procedure was performed separately for the S1 and S2 cortices to identify the response center in each region. Optical signals at the response center were used for analysis (Figures 2 and 3). The degree of response of each cortex was defined as the time-integrated value of the optical signal from each response center. The optical signal waveform obtained by binning 8 × 8 pixels (0.26 mm square) at the response center was used for the integral calculation (Figure 4).

For pseudo-color mapping of the cortical responses, the threshold level was determined to be above the fluctuation (baseline noise) of the optical signals before stimulation. The number of pixels mapped from the stimulation-activated area was counted frame by frame. After identifying the frame with the greatest optical signal amplitude, we determined the maximal activated area by calculating an average of five successive frames around the peak frame (Figure 3).

Statistical analyses were performed using Origin software (OriginLab Corp., Northampton, MA, USA). Data were analyzed using the Wilcoxon signed-rank test. In scatter plot analysis, regression lines were drawn, and goodness of fits were quantified according to coefficients of determination.

### 2.6. Histology

After the experiments, animals were deeply anesthetized with intraperitoneal pentobarbital and transcardially perfused with saline, followed by 4% paraformaldehyde in 0.1 M phosphate-buffered saline. A block of the brain, including the recorded cortical area, was carefully removed and postfixed overnight at 4 °C. Then, it was sectioned tangentially, and 80 µm sections were stained with cytochrome oxidase (CO) [15]. The pattern of vibrissa-related barrels was reconstructed from 2–3 CO-stained sections of layer IV to confirm agreement with optical imaging results [16].

## 3. Results

### 3.1. Optical Imaging of Whisker-Evoked Somatosensory Response

Cortical somatosensory responses after single-whisker stimulation were visualized using VSD and IS optical imaging. Figure 1A shows a surface image of the recorded cortical area and the layer IV barrel field confirmed after the experiments. The camera was positioned to include the S1 and S2 cortices in the field of view. Figure 1B shows the time course of optical signals recorded from each cortex. The VSD signal represents neural activity, and IS represents the consequent metabolic activity. Note that the time scales for the signals are very different. In the VSD signal, the S1 response showed an excitatory-inhibitory sequence of neural activity, while the S2 response showed long-lasting excitatory neural activity. Onset of the S2 response was delayed compared to the S1 response. Conversely, in IS, both S1 and S2 showed smooth bell-shaped changes, and the S2 response increased after the S1 response. Figure 1C shows VSD images demonstrating the spatiotemporal evolution of whisker-evoked neural activity. In S1, focal excitatory neural activity was observed in the corresponding barrel territory 12 ms after whisker deflection and spread to the surroundings; the excitatory activity disappeared by 60 ms, followed by the inhibitory neural activity. Meanwhile, in S2, excitatory neural activity appeared about 7 ms later compared to S1 and continued for about 150 ms. Figure 1D shows IS images demonstrating the spatiotemporal spread of metabolic activity. By 300 ms after whisker stimulation, metabolic activity appeared in the appropriate barrel in S1. Subsequently, the activity spread to the surrounding cortical areas and disappeared by 3000 ms. The metabolic activity in S2 was weaker, started about 300 ms later, and disappeared earlier compared to that in S1.

These results led to the following inferences. First, S1 responds before S2, suggesting a serial processing scheme of tactile stimuli in the rodent somatosensory cortex. Second, the inhibitory system is strongly involved in sensory processing in S1 but not in S2, suggesting that neural representation of sensory stimuli is markedly different between S1 and S2. Third, the energy demand of brain regions does not necessarily correlate with the degree of excitatory neural activity. Although excitatory neural activity was greater in S2 than S1, metabolic activity was greater in S1.

### 3.2. Onset Latency of Somatosensory Response

Figure 2 depicts the onset latency of the S1 and S2 responses recorded by VSD and IS imaging (n = 6). In VSD, the onset latencies were 7.6 ± 0.5 ms (mean ± standard error of mean, SEM) and 14.2 ± 0.5 ms, respectively, with a difference of 6.6 ms between the two regions. Conversely, in IS, the values were 294.4 ± 28.3 and 684.8 ± 62.4 ms, respectively. In addition, for both S1 and S2 responses, there was a correlation between the onset latency values obtained from the two imaging methods.

### 3.3. Amplitude and Extent of Somatosensory Response

To compare the characteristics of somatosensory responses obtained from VSD and IS imaging, we evaluated the maximum amplitude and cortical spread of the response and compared their ratio between S1 and S2. We only included excitatory neural activity in the analysis of VSD. Figure 3 shows the peak response ratio (S2/S1) and activated area ratio (S2/S1). The ratio of the maximum response amplitude of S2 to that of S1 was significantly different between VSD (1.098 ± 0.077) and IS (0.565 ± 0.054) (*p* < 0.05; Figure 3A, left panel). The scatterplot on the right shows the relationship between S2/S1 ratios obtained with the two imaging methods in individual animals, which suggests a good linear relationship between the two ratios (r^2^ = 0.70; Figure 3A, right panel). The areal ratio of maximum activation of S2 to that of S1 was significantly different between VSD (0.793 ± 0.075) and IS (0.432 ± 0.032) (*p* < 0.05; Figure 3B, left panel). A scatterplot of the relationship between the two ratios showed a linear relationship; however, the regression line did not have a good fit (r^2^ = 0.54; Figure 3B, right panel).

In Figure 3A,B, only responses mapped as excitatory neural activity are included in the VSD results. However, as shown in Figure 1C, excitatory neural activity was usually followed by inhibitory neural activity in the S1 response. Therefore, we separately evaluated the maximum activation area for each excitatory and inhibitory neural activity. The ratio of inhibitory to excitatory mapped region was 1.129 ± 0.067 (Figure 3C); inhibitory activity spread over a slightly larger cortical area compared to excitatory neural activity. In the next section, we explore inhibitory neural activity because it also contributed to the metabolic processes detected by IS imaging.

### 3.4. Degree of Somatosensory Response

Although peak amplitude and maximum activation area are simple indices for evaluating cortical responses, they lack information on temporal changes in sensory responses. Therefore, the degree of cortical sensory response was defined as the time integral of the optical signal (Figure 4A, top panel). The degree of sensory response for each cortex was determined using VSD and IS imaging, respectively, and the S2/S1 ratio was calculated. Figure 4A presents an analysis of the VSD signal without considering the downward (inhibitory) component. The bottom left panel shows the ratio of the degree of response of S2 to that of S1. The S2/S1 ratios were significantly different between VSD (3.224 ± 0.412) and IS (0.412 ± 0.06). A plot of the results obtained from individual animals showed a linear relationship between the two ratios (r^2^ = 0.57; Figure 4A, bottom right panel).

Figure 4A shows that the S2 excitatory neural activity was three-fold greater than that of S1; however, the associated metabolic activity was only about 0.4-fold that of (Figure 4A). This discrepancy may be because we did not take into account the inhibitory neural activity at the S1 cortex in the calculation of the degree of response. Therefore, next, we considered the inhibitory VSD component in the analysis. The VSD signal amplitude reflects the net sum of membrane potential fluctuation of a neuronal population. Thus, even if the signal amplitude is zero, it means that the depolarizing and hyperpolarizing membrane potentials of all of the neurons are balanced. Figure 4B1 shows an example of VSD imaging of the S1 response to whisker stimulation when the cortical inhibitory system is pharmacologically blocked. After administration of bicuculline, the time-series images show enhanced excitatory neural activity and reduced inhibitory neural activity. The red and green traces represent VSD signals before and after bicuculline, respectively; their difference (blue hatched area) can be considered the degree of response due to inhibitory neural activity. Based on this observation, we developed a simple model to estimate the degree of S1 response from the VSD signal showing an excitatory–inhibitory sequence (Figure 4B2). The top row shows a simplified waveform of the VSD response, usually observed in S1. We assumed that the waveform was obtained by adding the two waveforms shown in the middle (excitatory response) and lower (inhibitory response) rows. Accordingly, the total degree of response is the sum of the time integrals of the middle and lower waveforms, calculated using the recorded VSD signal and equation presented in the bottom square.

Figure 4C shows the same analysis of degree of response as Figure 4A, but the inhibitory neural activity is taken into account when determining the S1 degree of response (S1*_total_*) from the VSD data (top panel). S1*_total_* was derived by the method shown in Figure 4B2. The degree of response ratio S2/S1*_total_* was not significantly different between VSD (0.405 ± 0.051) and IS (0.412 ± 0.06) (*p* > 0.05; Figure 4C, bottom left panel). Compared to Figure 4A, the scatterplot shows a better linear relationship between the two ratios and better fit of regression line (r^2^ = 0.75; Figure 4C, bottom right panel). The similar degree of response ratio (S2/S1*_total_*) calculated from neural activity (VSD) and metabolic activity (IS) suggests that the metabolic demands of neural activity do not significantly differ between the S1 and S2 cortices.

## 4. Discussion

We performed VSD and IS imaging of whisker-evoked responses in S1 and S2 cortices of rats. First, for the S1 response, a sequence of excitatory–inhibitory neural activity was detected by VSD imaging, and a subsequent increase in metabolic activity was observed by IS imaging (Figure 1). These results are consistent with those reported previously [3]. However, this is the first study to compare the S2 responses obtained using two optical imaging methods. In VSD, the S2 response occurred later than the S1 response, and the S2 response showed prolonged excitatory neural activity, without the accompanying inhibitory neural activity seen in the S1 (Figure 1). It is unclear whether somatosensory information is processed from S1 to S2 in a serial or parallel manner. Human studies using fMRI have been inconclusive, with some supporting parallel processing [17,18] and others supporting serial processing [19,20]. By contrast, in nonprimate and lower primate mammals, tactile somatosensory processing is dominated by parallel processing through the networks between the thalamus and somatosensory cortices [21,22]. However, the onset latency difference of 6.6 ms between the S1 and S2 responses in the present study (Figure 2) suggests serial processing of tactile stimuli. The delay of a few milliseconds in the S2 response may be explained by signal transmission through cortico–cortical connections from S1 to S2 [23,24] or through the pathway from S1 to the posterior medial nucleus of the thalamus to S2 [25]. A recent VSD imaging study in mice also reported that the S2 response was delayed by about 3 ms from the S1 response following whisker stimulation [26]. VSD imaging in the present study and that by Hubatz et al. [26] showed that the S2 response was delayed, which contrasts with previous rodent studies that have reported similar onset latencies for S2 and S1 neurons [22,27]. This discrepancy may be because layer 4 neurons in S2 that receive thalamocortical inputs have sparse or absent apical dendrites in rodents [28], and VSD signals largely reflect membrane potential changes in dendrites in superficial layers [29]. Therefore, the initial response of layer 4 neurons in S2 to the thalamic input may be too small to be detected by the VSD signals. VSD imaging also showed strong inhibitory neural activity in S1 but not in S2 (Figure 1), which is useful for localizing sensory input in the primary sensory cortex [30]. Electrophysiological recordings from rats and mice show that the receptive fields of S2 to single-whisker stimulation are generally larger than those of S1 [31,32]. Furthermore, S2 has less temporal discrimination ability for repeated whisker deflections compared to S1, and neuronal discharges after a single stimulus are more dispersed in S2 than S1 [22,33]. These features of S1 and S2 responses may be explained by the differences in the strength of inhibitory neural activity in each region demonstrated by our VSD imaging results.

In the present study, IS imaging revealed a delayed onset of response in S2 compared to S1. In addition, the S2 response was weaker than the S1 response (Figure 1B,D). Although the difference in onset latency of neural responses between S1 and S2 was less than 10 ms (VSD imaging), the order of responses between the two regions was inherited in metabolic activity (IS imaging) and the time difference increased to about 300–400 ms (Figure 2A,B). This may be due to regional differences in hemodynamic lag between the S1 and S2 regions [34,35]. However, it is possible that the increase in the S2 response was small and gradual; therefore, its onset could not be accurately detected by IS imaging. Importantly, IS imaging showed a weaker S2 response compared to S1 response, which implies that the metabolic demand induced by whisker stimulation was less in S2 than in S1. However, this cannot be directly inferred from the VSD imaging results, which showed a stronger S2 response (Figure 1C,D). The ratio of the S2 to the S1 response was significantly greater in VSD imaging, both in the peak amplitude of the optical signal and in the activated cortical area (Figure 3A,B). Next, we calculated the degree of response as the time-integrated value of the optical signal but found similar results: S2 showed a greater degree of response compared to S1 in VSD imaging, while the opposite was true in IS imaging (Figure 4A). Assuming that VSD and IS imaging represent neural and metabolic activity, respectively, these results suggest that neurovascular coupling is different in the S1 and S2 regions, consistent with the findings from fMRI and local field potential recordings [9]. However, at this point, we do not consider the inhibitory neural activity detected by VSD imaging.

Recent optogenetic studies have shown that photostimulation of inhibitory neurons increases blood flow and arterial diameter, suggesting that inhibitory neurons significantly contribute to hemodynamic responses [36,37,38]. Therefore, it is essential to consider the contribution of inhibitory neural activity when calculating the degree of response. However, the VSD signal sums the membrane potential changes in the neuronal ensemble; therefore, it was not possible to identify individual excitatory and inhibitory responses from the recorded VSD signals [29]. To address this issue, we proposed a method to simplify the VSD signal waveform and decompose the excitatory and inhibitory response components. This model was inspired by the physiological experimental data shown in Figure 4B1. However, a similar model of neural activity has been used for fMRI data analysis [39]. In addition, the degree of response, defined as the time integral of the optical signals, was used to compare the sensory responses between cortical regions. In previous studies, we integrated optical signals to assess the degree of postsynaptic response masked by GABA_A_-mediated inhibition [40]. According to our proposed method, the ratio of the degree of response of S2 to S1 was similar, irrespective of whether the ratio was determined from VSD or IS imaging (Figure 4C). This suggests that neurovascular coupling does not vary between the S1 and S2 cortices, when the hemodynamic component resulting from inhibitory neural activity is taken into account. 

Our work had certain limitations that we must mention. First, optical imaging, especially VSD, is rather invasive. The extensive exposure of the brain surface and/or tissue staining with dyes may have affected brain function. Some voltage-sensitive dyes are associated with pharmacological side effects and modulation of GABAA receptor function [41,42]. The RH-795 dye that we used has not been reported to exhibit side effects affecting GABAA receptors, but caution should still be exercised when interpreting the hyperpolarizing signal component of the VSD signal. The slower hyperpolarizing signal component may reflect changes in both glial membrane potential and intrinsic hemoglobin signals. Therefore, future studies should evaluate simultaneous electrophysiological recordings, which would complement the results of VSD imaging and facilitate accurate interpretation. In addition, as the surface of the brain is curved, optical imaging of S1 and S2 may not have been uniform. Specifically, if the lateral S2 cortex was not perpendicular to the optical axis, IS imaging may have underestimated the S2 responses because of reduced light scattering. In terms of metabolic activity measurements, it would be useful to perform multiwavelength IS imaging when evaluating metabolic maps based on different hemodynamic components such as cerebral blood flow changes and variations in the levels of oxygenated and deoxygenated hemoglobin [43]. Moreover, the analytical model that we used to decompose excitatory and inhibitory responses is overly simplistic. We believe that this model needs further refinement and validation in the future. Alternatively, it would be possible to employ recently developed, genetically encoded voltage indicators to monitor the neural activities of targeted cell populations [44,45]; genetically encoded voltage indicator imaging enables fine distinction between excitatory and inhibitory neural responses. However, as conventional VSD imaging is convenient and will be used in many future studies, our analytical model should aid in the interpretation of imaging data.

In conclusion, previous studies that have combined fMRI and electrophysiology have failed to incorporate inhibitory neural activity into their analyses [8,9,10]; therefore, our combination of VSD and IS imaging is a new step toward identifying variation in neurovascular coupling among the brain regions.

## 5. Conclusions

This optical imaging study revealed differences in the cortical representation of sensory stimuli in S1 and S2 cortices. VSD imaging after whisker stimulation showed transient excitatory and subsequent inhibitory neural activity in S1 but prolonged excitatory neural activity in S2. IS imaging showed metabolic activity in the cortical region with neural activity, with S1 responses being greater than S2 responses. We proposed a method to evaluate the degree of neural and metabolic responses from the somatosensory cortex from VSD and IS optical imaging, respectively. The results showed that the ratio of the degree of response of S2 to S1 assessed by neural activity was similar to that assessed by metabolic activity, suggesting that neurovascular coupling did not vary between the S1 and S2 cortices. Although these results need to be further validated by refining the analytical model, the combination of VSD and IS optical imaging is a powerful method for investigating region-specific neural and metabolic responses and their coupling in the cerebral cortex.

## Data Availability

All data analyzed during this study are included in this published article.
